# Changes in γ-secretase activity and specificity caused by the introduction of consensus aspartyl protease active motif in Presenilin 1

**DOI:** 10.1186/1750-1326-3-6

**Published:** 2008-05-12

**Authors:** Donald B Carter, Edwige Dunn, Adele M Pauley, Denise D McKinley, Timothy J Fleck, Brenda R Ellerbrook, Nancy C Stratman, Xiangdong Zhou, Carol S Himes, Jeffrey S Nye, Alfredo Tomasselli, Riqiang Yan

**Affiliations:** 1 Global Research and Development, Pfizer Inc. Kalamazoo, MI 49001, USA; 2 Global Research and Development, Pfizer Inc. St Louis, MO 63017, USA; 3 Global Research and Development, Pfizer Inc. Groton, CT 06340, USA; 4 Department of Neurosciences, The Lerner Research Institute, Cleveland Clinic Foundation, Cleveland, OH 44195, USA; 5 St Louis Laboratories Pfizer Inc., 700 Chesterfield Parkway West, Chesterfield, MO 63017, USA

## Abstract

Presenilin (PS1 or PS2) is an essential component of the active γ-secretase complex that liberates the Aβ peptides from amyloid precursor protein (APP). PS1 is regarded as an atypical aspartyl protease harboring two essential aspartic acids in the context of the sequence D257LV and D385FI, respectively, rather than the typical DTG...DTG catalytic motif of classical aspartyl proteases. In the present studies, we introduced the sequence DTG in PS1 at and around the catalytic D257 and D385 residues to generate three PS1 mutants: D257TG, D385TG, and the double-mutant D257TG/D385TG. The effects of these changes on the γ-secretase activity in the presence or absence of γ-secretase inhibitors and modulators were investigated. The results showed that PS1 mutants having D385TG robustly enhanced Aβ_42 _production compared to the wild type (wt), and were more sensitive than wt to inhibition by a classical aspartyl protease transition state mimic, and fenchylamine, a sulfonamide derivative. Unlike wt PS1 and some of its clinical mutants, all three PS1 artificial mutants decreased cleavage of Notch S3-site, suggesting that these artificial mutations may trigger conformational changes at the substrate docking and catalytic site that cause alteration of substrate specificity and inhibition pattern. Consistent with this notion, we have found that NSAID enzymatic inhibitors of COX, known modulators of the γ-secretase activity, cause PS1 mutants containing D385TG to produce higher levels of both Aβ_38 _and Aβ_42_, but to reduce levels of Aβ_39_, showing a pattern of Aβ formation different from that observed with wild type PS1 and its clinical mutants. This study provides an important structural clue for the rational design of drugs to inhibit processing of APP at the γ-site without interfering with Notch processing.

## Introduction

Strong evidence supports the notion that the aberrant formation and accumulation of β-amyloid peptides (Aβ), generated from the β-amyloid-precursor protein (APP) by the action of β- and γ-secretase, is a critical event in the pathology of familial and sporadic forms of Alzheimer's Disease (AD) [1-3]. APP is initially cleaved by β-secretase to generate membrane-bound C-terminal fragments (CTFs), and a soluble N-terminal ectodomain sAPP_β _. Subsequently, those CTFs are cleaved by the γ-secretase within the putative transmembrane domain to release heterogeneous β-amyloid peptides (Aβ) composed of 37 to 43 amino acids; the 40 and 42 amino acids long peptides referred to as Aβ_40 _and Aβ_42_, respectively, are more predominant and the latter one is the most toxic to neurons [4]. Moreover, γ-secretase also cleaves APP at the ε-site (between L49 and V50), which resembles the γ-secretase S3 cleavage of Notch [5-8], and is located further downstream the γ-site with only 2–5 residues inside the cytoplasmic membrane [9]. Since the processing of Notch by the γ-secretase has important physiological implications, inhibition of its processing needs to be minimized in the development of γ-secretase inhibitors for AD therapy. Moreover, a variety of studies have already compiled a long list of potential γ-secretase cellular substrates that might further complicate selective inhibition of this enzyme. These substrates include CD44 [10], LRP [11], Erb4 [12], Nectin [13], E-cadherin [14]), and two membrane-bound ligands of Notch (Delta and Jagged) [15,16].

Both biochemical and genetic approaches have led to the discovery that at least four membrane-bound proteins, presenilin (PS1 or PS2), nicastrin, aph1 and pen2, are needed to form an active γ-secretase complex [17,18]. Interestingly, only mutations in PS1 and PS2 but not in the other γ-secretase components are a common cause of the early onset familial Alzheimer's disease (FAD) [3]. PS1 and PS2 FAD mutants have been shown to increase the ratio of Aβ_42 _over total Aβ species [19-22] by affecting γ-secretase activity through still poorly understood mechanisms. Notwithstanding, recent studies have demonstrated that a significant increase in absolute levels of Aβ_42 _was only observed for half of the mutations [23,24], further strengthening the notion that the mechanistic effects of PS1 mutations on the onset of FAD are complex.

Ample evidences suggest that PS1 (or PS2) is not only an indispensable component of a functional γ-secretase, but also is its catalytic core. In fact, complete deficiency of PS1 in mice abolishes the processing of both APP to release Aβ and Notch at the S3 site to release NICD [7,25]. Strikingly, mutations of the aspartic acid residues to Ala or Glu in transmembrane segment 6 (TM6) (D257) and TM7 (D385) in PS1 or PS2 (numbering according to PS1) result in a dramatic reduction of γ-secretase activity [26], implying the possibility of these two aspartic residues constitute the active site of the γ-secretase. Aspartyl protease inhibitors, such as L-685,458 [27,28] and difluoro ketones [29], were found not only to inhibit γ-secretase activity but also to locate or extract presenilins from cell homogenates by approaches including photoaffinity crosslink and affinity chromatography.

PS1 has a putative topology of nine-transmembrane domain (TM1 to TM9), and is matured and activated by cleavage between TM6 and TM7 by a highly regulated endoproteolytic cellular process that leaves behind tightly associated N- (NTF) and C-terminal fragments (CTF) [30]. Two conserved aspartic acids, D257 in TM6 and D385 in TM7, may potentially be positioned to face each other in an aqueous cavity within the lipid bilayer, and form the catalytic core [31]. Although mutation of either of these two conserved aspartic residues PS1 and PS2 impairs γ-secretase activity [32], they lack the two DTG (or DSG) triplets that form the catalytic pocket found in a typical aspartyl protease such as β-secretase [33] or HIV protease [34]. Similar to Presenilins, human signal peptide peptidase (SPP) possesses a pair of aspartic acids [35]. Moreover, SPP, PS1 and PS2 share highly conserved YD257 and GXGD385 (PS1 numbering) motifs and constitute a unique family of aspartyl protease [36].

While mutagenesis of residues surrounding the GXGD motif is reported [31,37,38], the sequence and structural contexts following these two essential aspartyl proteases are not understood. In this study, we artificially introduced either one DTG triplet or both DTG triplets in PS1 and asked whether the DTG triplet could alter γ-secretase catalytic property due to a structural resemblance to a classical aspartyl protease. We found that neither the single D385TG nor the double triplet D257TG/D385TG were able to convert PS1 into a classic aspartyl protease. However, D385TG in particular, dramatically elevated Aβ_42 _production, reinforcing the importance of residues surrounding D385 in the γ-secretase catalytic activity. More interestingly, PS1-D385TG significantly depressed the Notch S3-cleavage in releasing NICD, suggesting that the cleavage of APP at the γ-site and S3-site in Notch are differentially regulated. We also found that, unlike wt PS1 and other PS1 familial mutants, PS1-D385TG did not exhibit a biphasic profile on processing of APP in response to the inhibition by the γ-secretase inhibitors, L-685,458 or fenchylamine. We, therefore, propose a possible model in which PS1-D385TG adopts a conformation, within the γ-secretase complex, that differs from that of wt-PS1 and favors the production of Aβ_42 _but not NICD. This is the first demonstration that a mutation at F386I387 to TG causes such a dramatic effect in γ-secretase activity and inhibition. Because of its unique enzymatic profiles, PS1-D385TG will be valuable in a comparative structural exploration by approaches including electron microscope [39] as well as in rational design of drugs that specifically inhibit Aβ production.

## Results

### A DTG triplet in PS1 enhances Aβ_42 _production

In order to explore the potential catalytic effect of the aspartic acid residues in PS1 TM 6 and 7 domains, we generated mutant PS1 including those containing either a single DTG triplet or both triplets after residue D257 or/and D385 (see Table 1). The transient transfection experiments were carried out with equal amount of each mutated PS1 construct and secreted Aβ_40 _and Aβ_42 _were measured by ELISA. Two stable cell lines, named H125.3-16 and H167-11 cell lines that express human APP carrying Swedish or London mutation respectively, were used for assessing changes of secreted Aβ_40 _and Aβ_42_. The values of Aβ from the mock (empty pcDNA3 vector) transfected cells were used for normalization to calculate the % change in Aβ values obtained after transfection with the various plasmids shown in column 2. When the H125.3-16 cells were transfected with wild-type PS1 (wt-PS1), the ratio of Aβ_42 _over total Aβ was unchanged (Table 1). As expected, PS1 mutant carrying the familial mutation (PS1-M146V) [40,41] cause a preferential production of Aβ_42 _(Table 1). While mutant PS1 carrying a single DTG triplet at D257 (PS1-D257TG) behaved similar to wt PS1, mutant PS1 carrying a single DGT triplet at D385 (PS1-D385TG) caused a remarkably increased production of Aβ_42 _but not Aβ_40_, and this increase was even more dramatic than the above mutants (Table 1). A mutant PS1 containing both the M146V and D385TG mutations was not any more productive in Aβ_42 _secretion than either mutation separately (data not shown), suggesting no synergistic effect for these two mutations. Transient transfection of these PS1 mutant constructs into the human IMR-32 neuroblastoma cells or a mouse neuroblastoma cell line N2A-APP, which expresses human Swedish APP as previously described [42,43], also produced similar patterns of Aβ levels (data not shown). We also generated stable cells expressing both Swedish APP and various PS1 mutants as shown in Table 2. PS1 mutant harboring D385TG produced about 15-fold higher levels of Aβ_42 _than control while Aβ_40 _was only increased by 1.5-fold than control (Table 2). Similarly, stable cells expressing PS1-D257TG/D385TG also produced significantly higher levels of Aβ_42 _than control cells.

**Table 1:** Transient transfection of various PS1 constructs in HEK-293 cells derived stable cell lines expressing APP carrying Swedish mutation (H125.3-16).

**Cell Line**	**Transfected PS1 Constructs**	**% change Aβ1–40**	**% change Aβ1–42**	**ratio of Aβ42/Aβ**
H125.3-16	pcDNA	-	-	0.065 ± 0.004
"	PS1	-5.4	-8.6	0.063 ± 0.004
"	PS1-M146V	-10.5	33.7	0.095 ± 0.004**
"	PS1-D257TG	-5.4	-3.6	0.060 ± 0.003
"	PS1-D385TG	-2.7	233.7	0.193 ± 0.008*
"	PS1-D257TG/D385TG	-7.1	148.5	0.156 ± 0.001*

The levels of Aβ 40 and 42 were measured 48 hours post transfection.

** p < 0.05 as compared to PS1 wt transfected cells N = 3

* p < 0.01 as compared to PS1 wt transfected cells

Note: Due to the variation of absolute Aβ numbers in different experiments, only the comparative result to the cells transfected with pcDNA was used for comparison.

**Table 2:** Examination of Aβ values from conditioned media of HEK-293 derived stable cell lines expressing both Swedish APP and each of PS1 genes.

**Cell Line**	**Transfected PS1 Constructs**	**Aβ1–40 (pg/ml)**	**Aβ1–42 (pg/ml)**	**Ratio of Aβ_42_/Aβ**
H125.3-16	----	3152 ± 47 (n = 6)	337 ± 36 (n = 6)	0.097 ± 0.010
"	PS1	8379 ± 170 (n = 3)	1123 ± 20 (n = 6)	0.118 ± 0.002
"	PS1-M146V	9507 ± 129 (n = 3)	2627 ± 52 (n = 3)	0.217 ± 0.004**
"	PS1-D257TG	9716 ± 192 (n = 3)	1099 ± 35 (n = 6)	0.102 ± 0.003*
"	PS1-D385TG	7783 ± 225 (n = 3)	16647 ± 799 (n = 3)	0.681 ± 0.033**
"	PS1-D257TG/D385TG	5390 ± 218 (n = 3)	6941 ± 97 (n = 3)	0.563 ± 0.008**

n = 3 to 6 wells in one experiment. Aβ represents total Aβ.

*, p < 0.001, **, p < 0.0001 as compared to PS1 wt transfected cells

**Figure 1:**
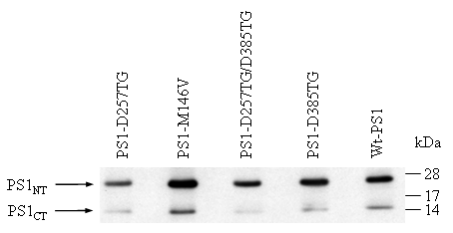
**Expression of PS1 variants in each stable cell line**. Western blot of equal amount of cell extracts from the stable cell lines expressing the indicated PS1 variants. The blot was probed with mixed antisera recognizing both N- and C-terminus of PS1.

A Western blot of protein extracts from transfected cells showed that transfected PS1 proteins in various cell lines are comparable (Figure 1), suggesting that the above shift of Aβ_42 _in cells expressing mutant PS1 was not due to the dramatically altered expression of PS1 variants. Thus, generation of artificial DTG motif at D385 appears to dramatically favor the production of Aβ_42_.

### The DTG triplet mimics PS1 familial mutation

Previously studies have shown that mutation of either D257 to A257 or D385 to A385 causes significant reduction of total Aβ production [26]. To determine whether the enhanced Aβ_42 _production seen in PS1-D385TG mutant was truly due to the introduction of an aspartyl protease DTG triplet, we then made substitutions to disrupt DTG motif in PS1-D385TG or PS1-D257TG/D385TG template. Interestingly, disruption of DTG motif in PS1-D257TA/D385TG, PS1-D257TG/D385TA or PS1-D385TA template did not cause reversion of the Aβ_42_/Aβ_total _ratio to the wt PS1 control (Table 3). Similar single mutation in BACE1 or HIV protease completely abolishes their proteolytic activity [44,45]. Thus, the presence of DTG triplet at D385 does not convert PS1 into a typical aspartyl protease, but rather creates a mutation that seems to resemble a PS1 mutation in FAD.

**Table 3:** Transient transfection of various PS1 constructs in HEK-293 cells derived stable cell lines expressing APP carrying Swedish mutation (H125-16).

**Cell Line**	**Transfected PS1 Constructs**	**Ratio of Aβ42/Aβ**
H125.3-16	PS1	0.082 ± 0.022
"	PS1-D385TG	0.261 ± 0.029**
"	PS1-D385TA	0.212 ± 0.007**
"	PS1-D257TA/D385TG	0.186 ± 0.010**
"	PS1- D257TG/D385TA	0.171 ± 0.005**

Aβ40 and Aβ42 levels were measured 48 hrs post-transfection.

** p < 0.01 (versus PS1 wt) N = 3

Changed residue in the DTG motif was shown in underlined.

### PS1 mutants display differential dose responses to γ-secretase inhibitors

**Figure 2:**
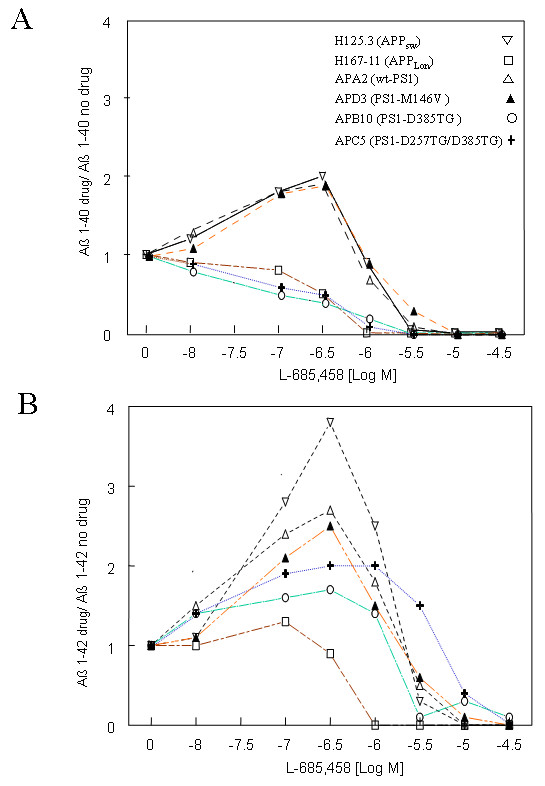
**The relative Aβ level secreted in the various stable cell lines treated with L-685,458.** Aβ_40 _(A) and Aβ_42 _(B) were measured by ELISA 24 hours post treatment with the γ-secretase inhibitor L-685,458 (n = 2). The untreated samples were used for normalization. H125.3-16 (APP-Sw, ▽), APA2 (APP-Sw + PS1, △), APB10 (APP-Sw + PS1-D385TG, ○), APC5 (APP-Sw + PS1-D257TG/D385TG, **+**), APD3 (APP-Sw + PS1-M146V, ▲) and H167-11 (APPV642F, □).

The effects on the secretion of Aβ, caused by the PS1 mutants harboring the DTG triplet were explored by treating transfected cells with two well characterized γ-secretase inhibitors: L-685,458, an aspartyl protease transition state analog [46], and fenchylamine, a sulfonamide derivative [47]. Production of Aβ_40 _was initially increased in APA2 cells (expressing both Swedish APP and wt PS1, △), APD3 (expressing both Swedish APP and PS1-M146V, ▲) and their parental line H125.3-16 (expressing only Swedish APP, ▼) upon addition of L-685,458 up to 0.3 μM, but became reduced with the increased dose of L-685,458 while reaching a complete inhibition at 3 μM (Figure 2A). The cell line APE12, expressing both Swedish APP and PS1-D257TG, showed a similar biphasic curve but in a lower dose range (0.1 μM and 1 μM, respectively; data not shown). Contrary to the above cases, cell lines expressing PS1 mutants PS1-D385TG (APB10,) and PS1-D257TG/D385TG (APC5, +) did not display biphasic curves and showed inhibition of Aβ_40 _production at all concentrations of the inhibitor used in the experiments. Interestingly, this non-biphasic curve was also seen in the cell line H167-11 expressing the London mutation APPV642F (□). This suggests that the conformational changes seen in APP London mutation are likely compatible with the D385TG mutation in PS1 during the interaction between the enzyme and its substrate; both facilitate γ-secretase to produce Aβ_42_.

The biphasic effects of L-685,458 on Aβ_42 _secretion from the cell lines expressing wt PS1 or familial PS1 mutation were even more robust than that on Aβ_40 _secretion (Figure 2B). Again, cell lines expressing either APP London mutation or D385TG triplet displayed no obvious biphasic effects. It appeared that L-685,458 has less inhibitory potency on Aβ_42 _production than on Aβ_40 _production as it required a higher dose to inhibit Aβ_42 _than Aβ_40 _in cells expressing PS1 carrying D385TG triplet. Cell toxicity was not obvious during the treatments with this drug up to 30 μM for any of the cell lines tested (data not shown), excluding the possibility that the Aβ reduction seen in the above experiments was due to drug toxicity.

**Figure 3:**
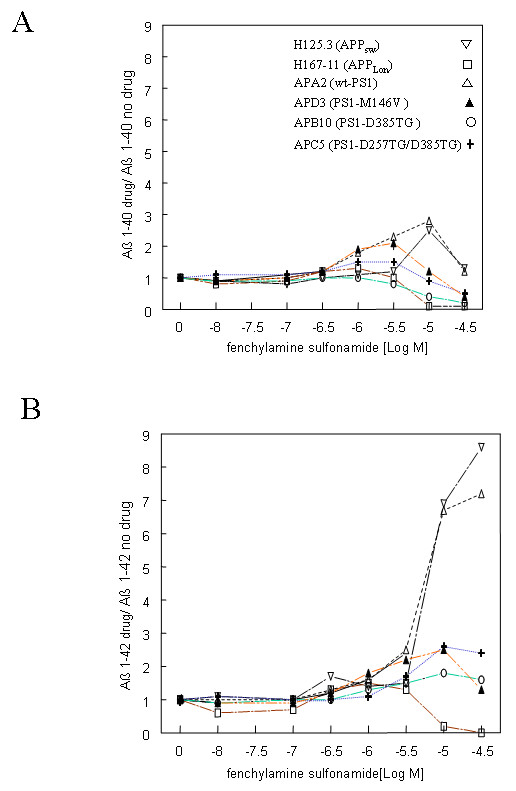
**The relative Aβ level secreted in the various stable cell lines treated with fenchylamine sulfonamide.** Aβ_40 _(A) and Aβ_42 _(B) were measured by ELISA 24 hours post treatment with the γ-secretase inhibitor fenchylamine sulfonamide (n = 2). The untreated samples were used for normalization. The symbol for each cell lines is the same as in Figure 2.

A biphasic production of Aβ_40 _was also seen in H125.3-16 (+), H143.3 (▽), and APA2 (△) cell lines treated with the less potent γ-secretase inhibitor fenchylamine sulfonamide: it took 12 μM to reach the peak of enzymatic activity and 30 μM to achieve complete inhibition (Figure 3A). Cells expressing PS1-D257TG (APE12) were also more sensitive to the drug treatment with 3 μM for the full stimulation (data not shown). Again, cells expressing PS1 D385TG mutants (APB10, ○) showed highest sensitivity to the treatment with non-biphasic responses (Figure 3A). Similarly, there appeared no biphasic Aβ_40 _production in cells expressing APP London mutation when treated with fenchylamine sulfonamide.

For the production of Aβ_42_, fenchylamine sulfonamide produced a large stimulus response on cells expressing endogenous PS1 or transfected wt PS1, but had a weak stimulating effect or no inhibition on the other cell lines (Figure 3B). The stimulating effect was probably due to the low potency of this drug on the inhibition of γ-secretase activity in producing Aβ_42_, and the biphasic effect reflects a low inhibitory profile.

### NSAIDs display differential effects on PS1 mutants

The nonselective COX inhibitors NSAIDs Ibuprofen, Indomethacin and Sulindac sulfide (an active metabolite of the pro-drug Sulindac) have been shown to reduce Aβ_42 _levels through increasing Aβ_38 _production [48], suggesting that NSAIDs can modulate γ-secretase activity. We found that all of the above compounds lowered Aβ_42 _secretion in a dose dependent manner in APB10 cell line expressing both Swedish APP and PS1-D385TG or APD cell line expressing both Swedish APP and the clinical mutation of PS1 M146V (Table 4). Sulindac sulfide was the most potent, followed by Indomethacin and Ibuprofen, showing 50% inhibition at about 30–50 μM for Sulindac Sulfide, 50–150 μM for Indomethacin and 300 μM for Ibuprofen. The nonselective COX inhibitors NSAIDs Aspirin (Acetylsalicylic acid) and Naproxen did not have any inhibitory effect (data not shown). The COX-2 NSAID inhibitor Meloxicam showed about 50% inhibition at 300 μM for both Aβ_40 _and Aβ_42 _which was similar to Ibuprofen for Aβ_42_, while Ibuprofen showed no effect on Aβ_40 _production.

**Table 4:** Effects of selected NSAID compounds on Aβ production from indicated cell lines.

Cell Lines	NSAID	Dose (μM)	Aβ_40 _(% of vehicle)	Aβ_42 _(% of vehicle)
APC5 cell line	(S) Ibuprofen	0	100	100
		33	101 ± 8	96 ± 17
		100	96 ± 15	79 ± 21
		300	70 ± 13 *	62 ± 9 *
				
	Indomethacin	0	100	100
		33	101 ± 11	79 ± 21
		100	93 ± 14	53 ± 12 *
		300	68 ± 11	11 ± 4 *
				
	Sulindac Sulfide	0	100	100
		11	103	88
		33	96 ± 5	64 ± 2 *
		100	57 ± 2 *	3 ± 1 *
				
APD3 cell line	(S) Ibuprofen	0	100	100
		33	135 ± 19	88
		100	119 ± 34	64 ± 2 *
		300	103 ± 36	31 ± 11 *
				
	Indomethacin	0	100	100
		33	95 ± 22	52 ± 15 *
		100	92 ± 12 *	13 ± 11 *
		300	72 ± 16 *	0 *
				
	Sulindac Sulfide	0	100	100
		11	111	76
		33	128 ± 18	56 ± 4 *
		100	64 ± 10 *	0 *

• * p < 0.05

• Note: at 11 μM Sulindac sulfide only one experiment was run which was the repeat experiment; due to the fact that this drug showed more potency than the others at lower doses that dose was added and the 300 μM dose was omitted since it exhibited toxicity on the cells

Each dose was run in triplicate.

**Figure 4:**
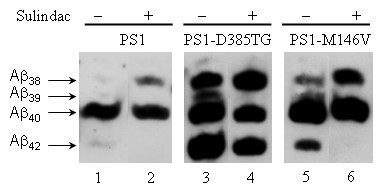
**Western blot of immunoprecipitated Aβ species.** Conditioned media collected from the cell lines that express wt PS1, PS1-D385TG or PS1-M146V and were treated with either DMSO or 100 μM Sulindac Sulfide. Equal amount of the media was immunoprecipitated with monoclonal antibody 4G8 and the extensively washed immunoprecipitates were resolved on a Tricine-Urea gel. The blot was reacted with antibody 6E10 for detection.

To examine the Aβ species under the treated conditions, media from the three tested cell lines expressing PS1, PS1-D385TG and PS1-M146V were collected, and Aβ peptides were immunoprecipitated with monoclonal antibody 4G8. Sulindac Sulfide at a concentration of 100 μM was chosen for treating the cell lines for its potent inhibitory effects seen in the above experiments. The immunoprecipitates were examined on bicine-urea gels to have better resolution of various Aβ species [48]. As shown in Figure 4, Aβ_40 _was a predominant species in this wt-PS1 expressing H125.3-16 cell line (Figure 4, lane 1). Aβ_42 _accounted for only 13% of total Aβ species that is defined as the sum of the Aβ 38, 39, 40, and 42 bands (as determined by optical density measurements). As expected, the H125.3-16 cells treated with Sulindac Sulfide produced no measurable Aβ_42 _while Aβ_38 _was increased from 15% to 33% of total Aβ species (Figure 4 lanes 2). Prior to any treatment, substantially more Aβ_42 _was produced in PS1-D385TG expressing cells (Figure 4 lanes 3) than in cells expressing either endogenous levels of wt PS1 (lanes 1) or PS1-M146V (lanes 5), and this was in consistent with the ELISA results. The Western blot results also indicated that PS1-D385TG elevated Aβ_38 _and Aβ_39 _production, and the increased Aβ_42 _species accounted for only 25% of the total Aβ in PS1-D385TG-expressing cells instead of 33% in PS1-M146V-expressing APC5 cells. After Sulindac Sulfide treatment, Aβ_42 _formation was completely shifted to Aβ_38 _in both H125.3-16 and APC5 cell lines (Figure 4, lanes 2 and 6). However, Aβ_42 _production was only partially reduced in PS1-D385TG-expressing cells under the same treatment conditions (Figure 4, lane 4). As noted, while the levels of Aβ_38 _were increased from 25% of total Aβ species to 33% in the PS1-D385TG expressing cells treated with 100 μM Sulindac Sulfide, it was the Aβ_39 _which had a more significant decline from 20% to undetectable in respect to the total Aβ species (Figure 4, lane 4). Apparently, NSAID has differential affects on the selective cleavage of APP-CTFs by the γ-secretase activity when PS1 is mutated.

### Differential effects on Notch S3-cleavage in PS1 mutants

**Figure 5:**
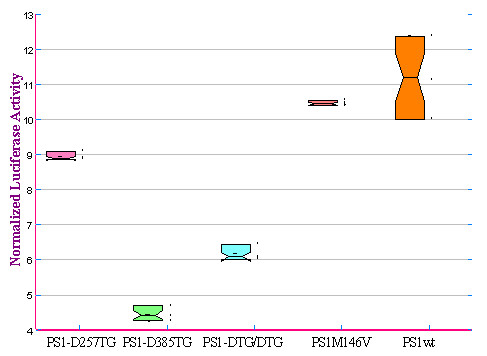
**PS1 carrying D385TG triplet suppresses NICD production**. Each indicated stable cell was transfected with the Notch ΔE-GVP, pFR-Luc and pRL-CMV. **In **Notch ΔE-GVP, the Notch ΔE protein was fused to VP16 transactivating protein domains (GVP). After the cleavage of the N ΔEGVP by the γ-secretase, the Notch ΔE-GVP intracellular domain will translocate to the nucleus and binds to the GAL4 DNA binding domain located in the upstream of the reporter pFR-Luc to activates transcription. The results are the average of the three experiments.

Notch receptors undergo three distinct proteolytic cleavages during maturation and activation, and the third cleavage of Notch (S3 site) occurs within the plasma membrane by the PS1-containing γ-secretase and results in the release and translocation of the intracellular domain into the nucleus to execute Notch signaling [49]. To determine whether the PS1 mutations carrying the DTG triplet would affect Notch processing, we developed a protocol based on the translocation of the Notch intracellular domain (NICD) to the nucleus after S3 cleavage [50]. For each of the 5 cell lines examined, cleavage of Notch by the γ-secretase activity was determined based on luciferase activity that measures binding of NICD to the reporter. Figure 5 summarized results from three independent experiments. While the clinical mutation PS1-M146V showed similar processing activity at the S3 site to the wild type PS1, a significant reduction of S3 cleavage was found in cells expressing PS1 mutants containing DTG motif at D385 (Figure 5). Specifically, the Notch cleavage activities of PS1-D385TG, PS1-DTG/DTG, PS1-D257TG and PS1-M146V have decreased by about 62%, 46%, 22% and 7%, respectively, compared to PS1wt activity (Figure 5). Obviously, the mutations in PS1 containing a DTG motif caused a significant change in the conformation in the γ-secretase complex that is not favoring the cleavage of the Notch substrate.

## Discussion

The triplets D257LV and D385FI, found in human PS1 TM 6 and 7, respectively, are conserved across species, and mutations of D257 and/or D385 to Ala result in essentially a complete loss of activity [26], suggesting that PS1 may function like an aspartyl protease. In addition, PS1 shares the YD257 and GxGD385 motifs with a unique family of aspartyl proteases referred to as signal peptidases (SPP) [35]. While the role of the amino acids comprising the YD257 and GxGD385 motifs have been studied, the couple of amino acids following D385, in particular F386, have not been tested. Since the two conserved catalytic aspartic acids D257 and D385 are not in the context of the two DTG (or DSG) catalytic triplets of a classical aspartyl protease [51], we artificially replaced L258V259 and/or F386I387 with TG in PS1 to generate two PS1 mutants D257TG and D385TG, and the double-mutant D257TG/D385TG; we then investigated the effect of these mutations on γ-secretase catalytic activity in the presence or absence of γ-secretase inhibitors. Our data shows that expression of the PS1-D257TG in a stable cell line Swedish APP did not significantly alter the production of Aβ_40 _or Aβ_42 _(data not shown). However, when the same experiment was carried out with PS1-D385TG or PS1-D257TG/D385TG, levels of Aβ_42 _were increased at least 2.5 fold compared to that with wt PS1 whereas Aβ_40 _remained largely unchanged. It appears that the mutation of F386I387 to T386G387 greatly favors the formation of Aβ_42_.

Equally interestingly, the F386I387 to T386G387 mutation results in a significant reduction in processing of Notch at the S3 site APP ε-site (ITL49↓V50ML) (Chen, 2001; Weidemann, 2002). Indeed, our preliminary experiments based on a reporter-depending assay suggested that both PS1-D385TG and PS1-D257TG/D385T also reduced processing at the APP ε-site (data not shown); yet, this latter observation needs to be fully validated. It appears that the mutation of F386I387 to T386G387 greatly favors the formation of Aβ_42 _and decreases the cleavage at both APP ε- and Notch S3-site.

We have shown that this enhanced production of Aβ_42 _in PS1-D385TG is not due to the conversion of PS1 into a classic aspartyl protease because the mutated triplet D385TA still increases production of Aβ_42 _to a certain extent (Table 3). In light of these and other findings (Tolia et al, 2006;Yamasaki et al., 2006), it appears that mutating I387 may have a minor effect on the γ-secretase activity. Instead, the effect is driven by the F386 to T386 mutation. The presenilin and signal peptidase consensus motif GxGD has been studied with regard to its role in the context of the γ-secretase activity. Interestingly, the clinical PS1 G384A mutant increased Aβ_42 _production by a factor of 6, but had no significant effect on Notch processing [52]. On the contrary, phenylalanine at position × of the GxGD385 motif in PS1 is suggested to be responsible for altering Notch processing [38]. Together with our findings that the mutation of F386 to T carries most of the responsibility for the observed change in substrate preference, we support the notion that the sequences surrounding the D385 may be critical in determining γ-secretase substrate recognition and docking, enzyme specificity and substrate cleavage rate. Although D257 is also implicated as a critical residue in cleaving APP at the γ-secretase site, PS1-D257TG has less effect on the elevation of Aβ 42 and reducing Notch cleavage in our experiment. This weak effect is likely due to the presence of wild type PS1 in our cell lines as suggested by others [53].

Consistently, PS1-D257TG and PS1-D385TG mutants were also affected differently by γ-secretase inhibitors in our studies when compared to the wild type or clinical mutants. Both wild type and most PS1 variants showed biphasic curves, stimulation at low inhibitor concentration followed by inhibition at higher concentration, to the γ-secretase inhibitor L-685,458 while the PS1-D385TG mutants showed normal dose-dependent inhibitory curves within the test range. This stimulation/inhibition behavior has been reported for a variety of apparently unrelated inhibitors, including aspartyl protease transition state inhibitors, both in cells and in cell-free assay of γ-secretase activity [54-57]. Indeed, increases of Aβ_42 _up to 8-fold were observed at the peak elevation, and Aβ 42 constituted as much as 50% of the total secreted Aβ [54]. Among the various mechanisms suggested to justify this phenomenon there is one involving an allosteric modulation of the γ-secretase complex by a process similar to that responsible for the increase of Aβ 42 production by FAD mutations in presenilins, possibly related to structural changes; and another one related to inhibitor binding to either PS allosteric sites or the cell membrane which would cause structural changes in the preseniln structure to favor Aβ 42 production prior to reaching inhibitory concentrations. No matter which stimulation/inhibition mechanism is operative, our PS1-D385TG mutant distinguishes itself from wild type and other mutants in that this mutation has changed the enzyme structure sufficiently to have a significant effect in its ability to recognize and cleave substrates and be inhibited. On the other hand, the mutation leading to PS1-D257TG has much less influence on inhibition than PS1-D385TG; yet it may be able to induce some minor structural changes since it is inhibited by lower concentrations of inhibitor than the wild type enzyme (data not shown).

It has been reported that the γ-secretase is composed of one of each of the following proteins: presenilin, nicastrin, aph1 and pen2 [58]. If this stoichiometry is correct, our results would favor a model which comprises a γ-secretase catalytic site with D257 and D385 as catalytic residues and F386 involved in substrate docking with other residues possibly from the GLGD385 motif. However, a different stoichiometry involving two molecules of presenilin per γ-secretase complex has been reported (Clarke et al. 2006; Schroeter et al, 2003). If the latter stoichiometry is true, an additional model could also be possible where two C-terminal fragments or two N-terminal fragments form a homodimer with two D385s or two D257s, respectively, as catalytic residues. Based on the larger effect exhibited by mutations around D385 than those produced by mutations around D257, we suggest that the mutations F386I387 to T386G387, particularly F386 to T, following the catalytic residue D385 in TMD7 of PS, are involved in APP/Notch substrate selection and in substrate docking at the active site of γ-secretase.

Earlier work [48] showed that, in cultured cells, certain NSAIDs were able to decrease Aβ_42 _secretion with concomitant increase in the Aβ_38_, suggesting a correlation between Aβ_42 _decrease/increase and Aβ_38 _increase/decrease. Recent work [59] has examined 10 clinical mutants of PS1 and shown that Sulindac Sulfide could increase the formation of Aβ_38 _without affecting Aβ_42_. Yet, these investigators observed that wt PS1 and even more PS1 M146L had a significant decrease of Aβ_42 _and increase of Aβ_38 _upon treatment with 50 μM sulindac sulfide. These latter observations are consistent, at least qualitatively, with those of the present study. Specifically, we show (Figure 4) that Sulindac Sulfide treated wt PS1, PS1 M146V, and PS1-D385TG, result in less Aβ_42 _production with a significant Aβ_38 _increase. Yet, while the two former proteins have a complete competence to produce Aβ_42_, the latter one has totally lost its ability to form Aβ_39_, and only partially that of making Aβ_42 _and Aβ_40_. The nature of these differences might be related to induction of conformational changes on these PS1 molecules by Sulindac Sulfide treatment [60] and are apparently different than those induced on most of the clinical mutants explored in the previous studies as reflected by the different effects on Aβ production [59].

In summary, we have, for the first time, demonstrated that a mutation at F386I387 to TG in PS1 causes a dramatic effect on γ-secretase activity with respect to higher productions of Aβ_42 _compared to wt PS1, and differential proteolytic cleavages of its two studied substrates by increasing cleavage of APP after residue 42 (yielding Aβ_42_) while decreasing the cleavage at the Notch S3-site. The knowledge gained from this study provides useful information in rational design and in the development of a protocol to screen compounds that only block the γ-secretase activity toward APP to release Aβ but not the other substrates. PS1-D385TG can be used as a tool for comparative studies of structure and conformation in the γ-secretase complex.

## Materials and methods

### Development of APP/PS1 Stable Cell Lines

HEK-293 cells were initially transfected with sixteen μg of DNA (pcDNA3.1/Hygro- vector inserted with either APP Swedish or London mutant. Two selected stable cell lines expressing Swedish APP were designated to be H143.3-23 and H125.3-16 while H167-11 for the cell line APP London mutant. A similar procedure was followed for the establishment of 125.3-16 cells that express PS1, PS1-D385TG, PS1-D257TG/D385TG, PS1-M146V, or PS1-D257TG cDNA inserts. The table below shows the nomenclature for the APP/PS1 stable cell lines and the clone used for follow-up studies. Three to ten clones were picked for each cell line and examined for the levels of Aβ_40 _and Aβ_42 _in conditioned media. The average ratio of Aβ_42_/total-Aβ for ten wt PS1-clones was 0.139 ± 0.22; 0.644 ± 0.089 for nine PS1-D385TG clones; 0.564 ± 0.133 for five PS1-D257TG/D385TG clones, 0.240 ± 0.033 for three PS1-M146V clones, and 0.087 ± 0.044 for ten PS1-D257TG clones. One clone for each PS1 DNA construct was selected from the set of clones for follow-up.

H125.3-16 APP-Sw

APA clone #2 APP-Sw + PS1 wt

APB clone #10 APP-Sw + PS1-D385TG

APC clone #5 APP-Sw + PS1-D257TG/D385TG

APD clone #3 APP-Sw + PS1-M146V

APE clone #12 APP-Sw + PS1-D257TG

### Notch Assays

Notch undergoes cleavage by the γ-secretase to release NICD that will translocate into nucleus to result in expression of target gene. To examine this cleavage, cell lines were seeded in 6 well dishes at 6 × 10^5 ^cells per ml (2 ml/well) and each well was transfected with 6.25 ng Notch ΔE-GVP [61], 1.55 μg pFR-Luc (UAS-firefly luciferase, Stratagene), and 62.5 ng pRL-CMV (renilla luciferase, Promega) for the luciferase assay or 2 μg Notch1ΔE for the Notch the next day. After incubation for 3 hrs, transfection media was replaced with growth media and cells were allowed to grow for an additional 48 hrs. Luciferase assay were performed according to the protocols from manufacturer (Dual-Luciferase Reporter Assay System, Promega). Briefly, each well was washed twice with PBS and harvested with 500 μl PLB buffer Lysates were transferred to eppendorf tubes and allowed to freeze at -80° for up to one week before being assayed. Lysates (2 or 20 μl) was transferred to each well of a 96 well plate and luciferase activity was measured using the Promega Dual-Luciferase reagents and a Lumiskan Ascent luminometer (ThermoLabsystems).

### ELISA Assay

The analysis of Aβ levels from conditioned media under specified conditions was performed as described previously [62]. Statistical analysis of the Aβ_40 _and Aβ_42 _levels was performed using the Student's t-test.

### Immunoprecipitation and Western blot Assay

Cells were first grown in DMEM media for 24 hours in 6 well dishes to near confluence and then treated with drugs such as 100 μM of sulindac sulfide in 1% DMSO. After incubation for 24 hrs, one ml of conditioned media was used for immunoprecipitation with monoclonal antibody 4G8 under standard overnight immunoprecipitation conditions as described previously [63]. The extensively washed immunoprecipitates were resolved on a Tricine-Urea gel. For Western with cell lysates, cell extracts were prepared in TENT buffer (50 mM Tris pH 8.0, 150 mM NaCl, 2 mM EDTA and 1% Triton X-100) with protease inhibitor cocktails. Equal amount of protein extracts were resolved on a 4–12% NuPage Bis Tris gel from Invitrogen (Carlsbad, CA). Monoclonal antibody 6E10 was used to detect Aβ species.

### Drug Treatment

Cells were plated at 50 to 100 thousand per well. After 48 hours, when cells were confluent, medium was replaced by cell medium containing the drug at each dilution. Each drug dilution was run in triplicate wells. After 24 hours incubation, half the volume of the conditioned cell medium was collected for measuring Aβ_40 _and Aβ_42 _by ELISA, while the remaining was saved for replication. The plate with the remaining cells was used for the MTS reduction assay to assess drug toxicity to the cells. All drugs were dissolved in DMSO at a concentration 1000 fold higher than the final drug concentration in the cell media for a final concentration of DMSO of 0.1%. The drugs used were Fenchylamine, L-685,458 (from Bachem), Sulindac sulfide and sulfone (a second metabolite of Sulindac) (all from Biomol Research Labs Inc.), Acetylsalicylic acid (from ICN), (S)-Naproxen (from Cayman Chemical Co), Meloxicam (from Calbiochem). The experiment with the selective γ-secretase inhibitors was replicated. The comparison was made between Aβ_40 _and Aβ_42 _levels after drug treatment versus mock treated control. Cellular toxicity in treated cells was evaluated according to the procedures as previously described [64].

## Competing interests

The authors declare that they have no competing interests.

## Note

Current address of the authors: Denise D McKinley is current at  Musculoskeletal Research, Eli Lilly and Company; 98C/B Drop Code 0403;  Indianapolis, IN; Jeffery S. Nye at CMO, East Coast Research and Early  Development, Johnson and Johnson Pharmaceutical R&D, LLC.  P.O. Box 776, Welsh & McKean Roads, Rm 31-2002 Spring House, PA. 
